# The Incidence of Peripheral Catheter-Related Thrombosis in Surgical Patients

**DOI:** 10.1155/2016/6043427

**Published:** 2016-01-24

**Authors:** Amy Leung, Clare Heal, Jennifer Banks, Breanna Abraham, Gian Capati, Casper Pretorius

**Affiliations:** ^1^James Cook University, School of Medicine and Dentistry, 4740 Mackay, QLD, Australia; ^2^Mackay Base Hospital, Mackay, QLD 4650, Australia; ^3^Mater Health Services, Corner of Raymond Terrace and Stanley Street, South Brisbane, QLD 4101, Australia

## Abstract

*Background.* Central venous catheters and peripherally inserted central catheters are well established risk factors for upper limb deep vein thrombosis. There is limited literature on the thrombosis rates in patients with peripheral catheters. A prospective observational study was conducted to determine the incidence of peripheral catheter-related thrombosis in surgical patients.* Methods.* Patients deemed high risk for venous thrombosis with a peripheral catheter were considered eligible for the study. An ultrasound was performed on enrolment into the study and at discharge from hospital. Participants were reviewed twice a day for clinical features of upper limb deep vein thrombosis during their admission and followed up at 30 days.* Results.* 54 patients were included in the study. The incidence of deep vein thrombosis and superficial venous thrombosis was 1.8% and 9.2%, respectively. All cases of venous thrombosis were asymptomatic. Risk factor analysis was limited by the low incidence of thrombosis.* Conclusion.* This study revealed a low incidence of deep vein thrombosis in surgical patients with peripheral catheters (1.8%). The study was underpowered; therefore the association between peripheral catheters and thrombosis is unable to be established. Future studies with larger sample sizes are required to determine the association between peripheral catheters and thrombosis.

## 1. Introduction

Upper limb deep vein thrombosis (DVT) refers to the formation of a fibrin clot within the subclavian, axillary, and brachial veins of the arm [1]. The vast majority of cases of upper limb DVT are associated with the use of indwelling intravenous catheters [2]. While any intravenous catheter has the potential to lead to venous thrombosis, the literature has mainly focused on the thrombosis rates of central venous catheters (CVCs) and peripherally inserted central catheters (PICCs) [3]. Over 330 million peripheral catheters are placed every year in America alone, required by up to 70% of patients in hospital. Peripheral catheters are often used in surgical patients for the administration of intravenous fluids and intravenous medications and for anaesthetic purposes [4]. Although the insertion of a peripheral catheter is the most common invasive procedure in hospitals, there is a paucity of data regarding thrombosis rates in patients with peripheral catheters. As many surgical patients require a peripheral catheter during their admission, we have conducted a prospective study to detect the incidence of peripheral catheter-related DVT in the general surgical unit and the potential associated risk factors. A secondary aim was to determine the incidence of catheter-related superficial vein thrombosis (SVT).

## 2. Methods

### 2.1. Eligible Patients

All patients hospitalized in the general surgical unit classified as high risk for venous thromboembolism (VTE) with a peripheral intravenous catheter in the dorsal hand or forearm were considered for inclusion. We accepted peripheral intravenous catheters of any gauge. We defined high risk as age over 65 years and one other risk factor either an underlying malignancy, thrombophilia (Factor C deficiency, Factor S deficiency, Factor 5 Leiden mutation, antithrombin 3 deficiency, antiphospholipid syndrome, and prothrombin 20210A mutation), or recent major surgery according to criteria from the Royal Australasian College of Surgeons (RACS) [5]. Exclusion criteria included previous or current upper limb or chest trauma, previous or current upper limb, neck, or axillary surgery, previous or current upper limb DVT, previous mastectomy, thoracic outlet syndrome, enlarged lymph nodes/tumours causing compression, or current central venous catheter or peripherally inserted central catheter. Participants were prospectively followed up for 30 days from enrolment.

### 2.2. Study Design

The study was a prospective observational single arm cohort study conducted in the general surgical unit of Mackay Base Hospital. The study period occurred from August 2014 to July 2015. The study received ethical approval from the Townsville Hospital and Health Services Human Resources and Ethics Committee on the 30th of May 2014. Informed consent was obtained from all individual participants included in the study. On enrolment, the participant's demographics, medical comorbidities, full blood count, international normalised ratio of prothrombin time of blood coagulation (INR), and catheter characteristics were collected. All patients had an ultrasound examination of the superficial and deep veins of the cannulated arm on enrolment into the study and at discharge. Colour duplex sonography was selected for diagnosis of DVT as it has a high sensitivity (93%) and specificity (93%) and is noninvasive and easy to perform [6]. All ultrasounds were performed by the sonographers at the Mackay Base Hospital Radiology Department. The criteria for upper extremity thrombosis were defined as the presence of a thrombus, lack of flow, nonpulsatile and nonphasic flow, and lack of compressibility of the veins [7]. DVT was classified as thrombosis of the subclavian, axillary, or brachial veins. Thrombosis of the cephalic or basilic vein was classified as a SVT. During the admission period, patients were reviewed twice a day for signs and symptoms of upper limb DVT. Patients were asked if they had experienced upper limb pain and swelling. On examination, abnormal vital signs, tenderness, oedema, and erythema of the cannulated arm were noted. All patients were followed up at thirty days with either a phone consult or outpatient appointment to review symptoms of venous thromboembolism (VTE).

### 2.3. Statistical Analysis

The incidence of peripheral catheter-related DVT has only been reported in one previous study. Therefore we estimated a 20% prevalence based on the results of this study. Based on the estimated prevalence, we calculated a sample size of 62 participants for 95% Confidence Intervals (CI) of 10% to 30%. The incidence of peripheral catheter-related thrombosis is reported as the proportion of enrolled patients who had an ultrasound confirmed thrombus. Corresponding 95% CIs were calculated for the proportions of thrombosis. Descriptive statistics were generated for all included cases. Continuous variables were expressed as medians and interquartile ranges (IQR 25th and 75th percentile) or means and standard deviations (SD). The categorical variables were expressed as numbers and percentages. Bivariate analysis was performed using Fisher's exact test with thrombosis as the dependent variable and various risk factors as the independent variable. The data was analysed using SPSS version 20 (SPSS Inc., Chicago, IL, USA) for Windows; statistical significance was given to *P* values < 0.05.

## 3. Results

From August 2014 to July 2015 there were 337 participants aged 65 years and over who had a peripheral catheter placed in the dorsal hand or forearm in the general surgical unit. Two-hundred and sixty patients did not meet eligibility criteria and 17 declined to enter the study. Sixty patients consented to participate in the study. Six patients did not complete the study. Five patients were lost to follow-up and one patient passed away from an underlying medical condition unrelated to the study. In total, data from 54 patients was analysed. The participant characteristics are listed in Table 1. None of the recruited participants had a thrombophilia. The incidence of upper limb DVT was 1.8% (1 out of 54 patients, 95% CI −1.7% to 5.4%). The thrombus was located in the brachial vein. The only risk factors the patient had were age over 65 years and recent surgery. The patient was commenced on six months of warfarin as per the physicians at Mackay Base Hospital. The incidence of SVT was 9.25% (5 out of 54 patients, 95% CI 1.5% to 17%). Four were located in the cephalic vein and one in the basilic vein. All patients with SVT were treated with 30 days of therapeutic low molecular weight heparin (LMWH) as per the American College of Chest Physician Guidelines for antithrombotic therapy for venous thromboembolic disease [8]. The characteristics of the participants with peripheral catheter-related venous thrombosis is displayed in Table 2. The overall incidence of thrombotic events was 11.1% (95% CI 2.7% to 19.5%). All cases of venous thrombosis were asymptomatic. There were no cases of clinically detected pulmonary embolism. None of the risk factors investigated were found to be statistically significant for association with upper extremity thrombosis, as listed in Table 3. At the 30-day follow-up there were no reported adverse events.

## 4. Discussion

Ours is the first prospective study designed to detect the incidence of peripheral catheter-related DVT and to identify associated risk factors in surgical patients. We detected a low incidence of peripheral catheter-DVT (incidence of 1.8%) with no associated risk factors. However there was a higher incidence of peripheral catheter-related SVT (9.8%). All cases of peripheral catheter-related thrombosis in our study were asymptomatic and detected on ultrasound as a result of our study. There were no cases of pulmonary embolism clinically detected in our study.

The low incidence of peripheral catheter-related DVT reinforces the results of a previous study. A randomised control trial comparing the efficacy of PICCS and peripheral catheters found a 3.4% incidence of peripheral catheter-related DVT. Similar to our study ultrasound was performed at the beginning and end of catheterization. There was a higher incidence of peripheral catheter-related SVT than the incidence of peripheral catheter-related DVT in our study. The incidence of peripheral catheter-related SVT was lower than figures reported in a previous study (9.2% compared to 44%). The increased incidence of SVT in Periard's study is likely attributed to larger gauge of catheters used and increased time of catheterization [9]. The thrombogenicity of peripheral catheters can be attributed to endothelial injury and venous stasis, two of the components of Virchow's triad of thrombosis. The endothelium is often damaged during the insertion of a peripheral catheter [3]. In addition, the presence of a peripheral catheter in a vein reduces blood flow and potentially causes venous stasis. Peripheral catheters are often inserted into the smaller superficial veins of the upper limb creating a smaller catheter-to-vein ratio. Catheter-to-vein ratio has been suggested to be a contributing factor to thrombosis due to increased risk of stasis from a slower flow. This is evident in studies with PICC-lines as PICCs inserted into the smaller cephalic veins are more likely to thrombose [10].

We assessed recent surgery, malignancy, metastatic disease, increased BMI, diabetes mellitus, cannula gauge, number of attempts at cannulation, and length of catheterization for association with catheter-related thrombosis. None of the risk factors assessed in our study were found to have a significant association with catheter-related thrombosis. However, the data analysis was limited by the small number of events. In our study 83% of participants with upper extremity thrombosis had recent surgery. Previous studies report mixed results regarding recent surgery as a risk factor for catheter-related thrombosis [11–15]. Wilson et al. demonstrated that surgery >1 hour in length was a risk factor for catheter-related thrombosis in a prospective study. The development of a thrombus was attributed to venous stasis from prolonged paresis of the arm during surgery [15]. Half of the patients with a peripheral catheter-related thrombosis had a malignancy. The association between malignancy and venous thrombosis has been well established. Malignancy is believed to induce a hypercoagulable state through activation of the coagulation cascade and generation of thrombin [11].

The results of our study raise questions regarding screening for asymptomatic upper extremity thrombosis. None of the patients with peripheral catheter-related thrombosis were symptomatic. Previous studies have reported similar findings where all cases of thrombosis were found incidentally on ultrasound [9]. These results confirm the need for imaging in the diagnosis of peripheral catheter-related thrombosis as most cases are asymptomatic.

There are strong arguments in literature regarding anticoagulation of symptomatic upper limb DVT as it prevents propagation of the thrombus and development of a pulmonary embolism (PE) [9]. The clinical significance of an asymptomatic upper limb DVT is poorly documented as the majority of studies are conducted on symptomatic patients [16]. As the nature of asymptomatic upper limb DVT is unknown, the risks and benefits of treatment are unknown. Theoretically the risk of embolization of the asymptomatic thrombus should be the same as a symptomatic upper limb DVT. In fact, asymptomatic DVT of the lower limb has long been regarded as a source of embolization. In a large autopsy series lower limb DVT was detected in 83% of patients who died from PE; however only 19% were symptomatic [17]. The findings of the study suggest that asymptomatic upper limb DVT may be an overlooked source of PE. However large randomised studies would be required to determine the clinical significance of an asymptomatic upper limb DVT.

We detected five cases of SVT in our study. SVT has previously been regarded as a benign disease; however there is a growing body of evidence to suggest otherwise [18, 19]. Decousus et al. demonstrated that 25% of patients with a lower limb superficial venous thrombosis developed a concomitant deep vein thrombosis and 3.9% had a symptomatic pulmonary embolism [20]. While there are currently no studies regarding the clinical significance of an upper extremity DVT, based on the studies conducted on the lower limb we decided to treat patients diagnosed with SVT with 30 days of LMWH. At the 30-day follow-up there were no adverse events or complications reported.

There were no cases of clinically detected PE in our study. Similar findings have been reported in previous studies. In a large retrospective series, Chemaly et al. reported an incidence of 3.4% of PE in patients with PICC-lines [21]. The incidence of asymptomatic PE is higher in patients with catheter-related thrombosis, with up to 17% of patients with catheter-related thrombosis having an asymptomatic PE [22]. However the mortality rate associated with asymptomatic PE appears to be low. It is uncertain whether investigation of asymptomatic PE would be of benefit considering the invasiveness of the investigations required [23].

There are several limitations to our study. The present study is a prospective observational study conducted in a single regional institution; therefore the findings of this study may not be applicable to other hospital populations. There is inherent bias associated with the observational design of the study as confounders are unable to be controlled and there are a lack of internal controls. The study was underpowered; therefore the confidence intervals are very broad, indicating that the results are imprecise. The low incidence of peripheral catheter-related thrombosis limited the ability to identify associated risk factors. Nonconsecutive patients were enrolled into the study; therefore the study sample may not be a true reflection of the intended sample. Ideally, both arms would be scanned for a DVT; however this would have been too difficult considering financial constraints of the study. Furthermore patients were only followed up for 30 days; therefore symptoms of upper limb DVT after 30 days are unknown.

Despite the limitations, there were also several strengths with this study. This is the first study to investigate the incidence of peripheral catheter-related venous thrombosis in surgical patients. The prospective design and investigation of all study participants prevented missing any case of peripheral catheter-related thrombosis. In addition this confirmed the asymptomatic nature of peripheral catheter-related thrombosis. We also used objective investigations to diagnose thrombosis preventing misdiagnosis. This is also the first study to investigate the risk factors for upper limb superficial venous thrombosis.

## 5. Conclusion

In our study we recorded a low incidence of peripheral catheter-related DVT (1.8%, 95% CI −1.7% to 5.4%), but a higher incidence of superficial venous thrombosis (9.25%, 95% CI 1.5% to 17%). The small sample size and low incidence of peripheral catheter-related thrombosis limited our ability to determine the association between peripheral catheters and upper limb DVT. Future studies will be required to show the natural history of an asymptomatic upper limb DVT through regular ultrasounds and investigation for occult pulmonary emboli.

## Figures and Tables

**Table 1:** Characteristics of the participants.

Variable	Result
Age (years), median (IQR)	73 (69–79.25)
Gender, *n* (%) female	21 (38.9%)
Ethnicity, *n* (%)	
Caucasian	53 (98.1%)
Aboriginal and Torres Strait Islander	1 (1.98%)
Anticoagulation, *n* (%)	25 (46.3%)
Haemaglobin, median (IQR)	130 (121.75–142.25)
Platelets, median (IQR)	226 (177–316.5)
INR, median (IQR)	1.1 (1.0–1.1)
Catheter size, *n* (%)	
18 G	48 (88.9%)
20 G	3 (5.6%)
22 G	3 (5.6%)
Time of catheterization in days, median (IQR)	3.83 (2–5)
Location of peripheral catheter, *n* (%)	
Dorsum hand	17 (31.5%)
Wrist	6 (11.1%)
Forearm	10 (18.5%)
Cubital fossa	21 (38.9%)
Resited peripheral catheters, *n* (%)	52 (96.3%)
Number of attempts at catheterization, mean (SD)	1 (0.79)
BMI, *n* (%)	17 (31.5%)
Diabetes, *n* (%)	15 (27.8%)
Malignancy, *n* (%)	20 (37%)
Metastatic disease, *n* (%)	3 (5.6%)
Recent surgery, *n* (%)	40 (74.1%)

**Table 2:** Characteristics of the participants with upper extremity thrombosis.

Age	Sex	Location of catheter	Size of catheter	Risk factors	Clinical signs	Type of thrombus	Thrombus location	Size of thrombus	Treatment	Symptoms at 30 days
71	F	L forearm 5 cm from wrist	20 G	Recent surgery, type 2 DM, and obesity	No signs	SVT	Cephalic vein → 5 cm from cannula site	Speck	LMWH	No symptoms

85	F	L cubital fossa	20 G	Colorectal cancer, recent surgery	No signs	SVT	Cephalic vein	7 cm segment of thrombus	LMWH	No symptoms

72	F	L cubital fossa	20 G	Bladder lymphoma	No signs	SVT	Cephalic vein	Thrombus at site of cannula	LMWH	No symptoms

81	F	L cubital fossa	20 G	Colorectal cancer, recent surgery	No signs	SVT	Cephalic vein	Segment of thrombus	LMWH	No symptoms

83	M	R dorsal hand	22 G	Recent surgery	No signs	DVT	Brachial vein	Segment of thrombus	Warfarin	No symptoms

70	M	L cubital fossa	20 G	Recent surgery	No signs	SVT	Basilic vein	Nonocclusive	Warfarin	No symptoms

**Table 3:** Risk factors.

Risk factor	Number of thromboses	*P* value
Gender (female)	4/21 (19%)	0.193
Ethnicity (Caucasian)	6/53 (11.3%)	1
Diabetes	1/15 (6.66%)	1
Metastatic disease	0/3 (0%)	1
Anticoagulation	3/25 (12%)	1
Elevated WCC	1/8 (12.5%)	1
BMI >30	2/17 (11.76%)	1
Cannula LOS >1		NS
Size of cannula		NS
Cubital fossa	4/21 (19%)	NS
More than 1 attempt	1/12 (8.33%)	NS
Malignancy	3/20 (15%)	0.659
Surgery	4/40 (10%)	0.643
